# Maternal Thyroid Function During Pregnancy and the Child’s Linguistic and Sensory Development in the Northern Finland Birth Cohort 1986

**DOI:** 10.3389/fendo.2018.00127

**Published:** 2018-03-26

**Authors:** Fanni Päkkilä, Tuija Männistö, Anna-Liisa Hartikainen, Eila Suvanto

**Affiliations:** ^1^Department of Obstetrics and Gynecology, University of Oulu, Oulu, Finland; ^2^Clinic of Obstetrics and Gynecology, Oulu University Hospital, Oulu, Finland; ^3^Institute of Health Sciences, University of Oulu, Oulu, Finland; ^4^Department of Children, Young People, and Families, National Institute for Health and Welfare, Oulu, Finland; ^5^Medical Research Center Oulu, Oulu University Hospital, University of Oulu, Oulu, Finland; ^6^Department of Chronic Disease Prevention, National Institute for Health and Welfare, Oulu, Finland; ^7^Northern Finland Laboratory Center Nordlab, Oulu University Hospital, University of Oulu, Oulu, Finland; ^8^Department of Clinical Chemistry, University of Oulu, Oulu, Finland

**Keywords:** sensory development, linguistic abilities, hypothyroidism, epidemiology, pregnancy, thyroid disorders

## Abstract

**Background:**

Maternal hypothyroidism and hypothyroxinemia are associated with poor neuropsychological development in children. Previous research is lacking on whether maternal thyroid dysfunction affects sensory and linguistic development in childhood.

**Methods:**

The Northern Finland Birth Cohort 1986 included all births within a year (9,362 women, 9,479 children) from the two northernmost Finnish provinces. Maternal serum samples (*n* = 5,791) were obtained in early pregnancy and analyzed for TSH, free T4, and thyroid peroxidase antibodies (TPO-Abs). Five thousand three hundred and ninety-one parents evaluated their child’s sensory and linguistic development at 7 years old *via* a questionnaire (excluding children with an intelligence quotient ≤85). The prevalence of sensory and linguistic impairments was compared between mothers with and without thyroid dysfunction.

**Results:**

There were no statistically significant differences in the prevalence of sensory or linguistic impairment between children of mothers with and without thyroid dysfunction. Children of hypothyroid and hypothyroxinemic mothers had an increased prevalence of vision impairment compared with those of euthyroid mothers (10.8 and 11.7%, respectively, versus 6.5%), but the difference was not significant. All results remained similar after excluding TPO-Ab-positive mothers and premature children.

**Conclusion:**

We did not find an association between maternal thyroid dysfunction during pregnancy and sensory and linguistic development impairment in childhood. A somewhat higher prevalence of vision impairment was seen in children of hypothyroid and hypothyroxinemic mothers, which merits further research.

## Introduction

Pregnancy stresses the thyroid gland, and overt hypo- and hyperthyroidism are considered significant risk factors for pregnancy complications and adverse childhood development (1–3). Large population-based cohort studies have suggested that maternal subclinical hypothyroidism and hypothyroxinemia and/or thyroid peroxidase antibody (TPO-Ab) positivity might also adversely affect offspring’s neuropsychological development (4–7) and psychiatric morbidity (8, 9).

The fetal brain needs thyroid hormones (THs) even before the onset of fetal TH production, which occurs around 20 weeks gestation. Thus, the fetus is dependent on maternal TH supply (10). THs regulate genes responsible for neurogenesis, cell migration and differentiation, synaptogenesis, and myelination (11–14). Rat studies have shown that maternal THs are important for the offspring’s sensory development. Maternal THs affect several structures needed for normal vision, such as the retina (15), thalamus (16), and primary visual cortex (17).

In humans, insufficiently treated maternal overt hypothyroidism during early pregnancy or congenital hypothyroidism in the child reduced infant’s contrast sensitivity (18). Also, children who developed transient hypothyroxinemia of prematurity were at a greater risk of impaired visual functioning (19) and psychomotor delays (20) compared with prematurely born infants without transient hypothyroxinemia of prematurity. These studies emphasize the need of THs in humans during fetal development and in early life.

Some previous studies have examined the sensory and linguistic development in children of mothers with thyroid dysfunction with mostly negative findings (21–24). However, these studies were limited by short follow-up times and limited sample sizes as the sensory and linguistic developmental outcomes were secondary outcomes. In contrast, language development was studied as the main outcome in one large cohort study, which showed an association between maternal hypothyroidism during pregnancy and children’s expressive language delay (25). Additionally, maternal TPO-Ab-positivity in the third trimester has been associated with an increased risk of childhood sensorineural hearing loss by 8 years old (26). In adults, treating hypothyroidism with levothyroxine improves color contrast sensitivity (27).

With this background, we hypothesized that maternal thyroid dysfunction and especially maternal hypothyroidism during pregnancy might negatively affect the child’s visual, linguistic, and audiological development. We tested the hypothesis in the prospective, population-based, and iodine-sufficient Northern Finland Birth Cohort 1986 (NFBC 1986) by analyzing parental questionnaire data and maternal early pregnancy laboratory data.

## Materials and Methods

### Northern Finland Birth Cohort 1986

The prospective NFBC 1986 covered 99% of all births with a calculated term between July 1, 1985 and June 30, 1986, drawn from the two northernmost provinces of Finland (9,362 mothers, 9,479 children). Maternal and family demographics, maternal health data, and data on pregnancy, delivery, and neonatal outcomes were collected during routine visits to free communal maternity welfare clinics (participation rate 99.8%) and *via* questionnaires during the index pregnancy. All mothers’ follow-up started at the first visit to a clinic at 8–12 weeks gestation (28, 29). They were recruited to the study at 24 weeks gestation.

After birth, data on the health of cohort children and familial demographics were obtained *via* visits to the free communal child welfare clinics, questionnaires, and clinical examination, supplemented with data from national registers (29). All subjects provided informed consent. The Ethics Committees of the Northern Ostrobothnia Hospital District and the National Institute for Health and Welfare approved this study.

### Data Collection

#### Detection of Developmental Problems

In Finland, all children participate in a follow-up protocol in the local communal child welfare clinic. This practice is regulated under the Finnish Law on Public Health Care [1326/2010 (Finlex)]. The purpose of the follow-up program is to detect early-stage developmental delays and enable early treatment (30). The percentage of non-participating families is approximately only 0.2–0.3% (Register data from the National Institute for Health and Welfare, Finland).

The NFBC 1986 children received clinical examinations since birth according to the national program, from either a doctor or a trained nurse, approximately once a month for 1 year. After the first year, the clinical examinations continued once or twice a year depending on the family’s needs.

Children’s hearing was examined at ages of 8 months, 4 and 6 years according to the follow-up program. The children’s pupillary reflexes and strabismus were studied at every doctoral visit (at 1, 6, 8, 12, and 16 months). After that, the children’s vision was examined once a year. The children’s speech development was routinely assessed at every visit to a nurse or doctor (30). The children were additionally examined at any age if parents expressed a concern about abnormal hearing, speech, or visual development.

#### Parental Questionnaires About 7-Year-Old Children

When the children were 7 years old and in their first year of school (*n* = 9,326 children alive and with a known address), the parents were sent the first of two postal questionnaires (response rate 90.2%). The questionnaire included questions on school and family type, family’s social environment, and the child’s development and health. The specific sensory and motor development questions were designed in a way that the parents could answer them based on the routine child follow-up program examinations. The questionnaire included a question about vision deficits. If a deficit was present, the parents were queried about its nature (squinting, refractive errors, or other). Abnormal hearing was evaluated similarly. The parents also evaluated if the child had abnormal faculty or development of speech. They were asked about the presence of dysphasia or need for speech therapy until the age of four. The study protocol and the questionnaire form were previously reported in more detail (31).

#### Maternal Thyroid Function During Pregnancy

The NFBC 1986 mothers underwent routine infectious disease screening during the first trimester at their first visit to a local maternity welfare clinic. The mean gestational age was 10.7 weeks (SD 2.8) at sampling. The remainder of the serum samples was stored in the Finnish Maternity Cohort at −25°C on the grounds of the National Institute for Health and Welfare (32). The available samples with sufficient serum size (*n* = 5,805, 61.2% of the NFBC 1986) were analyzed for TSH, fT4, and TPO-Abs (or for TSH and/or fT4 if sample size was insufficient for all analyses) with the Abbott Architect i2000 method (Abbott Diagnostics, Abbott Park, IL, USA). Five thousand seven hundred and ninety-one samples were sufficient for TSH and fT4 analysis. Data on laboratory data collection (33) and the long-term storage effects of these laboratory parameters were published previously (32). Maternal demographic characteristics and birth outcomes of mothers with and without available laboratory parameters were similar (32). A medical chart review revealed 98 women with diagnosed and treated thyroid disease. Sixty-eight mothers needed thyroid medication during gestation or had used thyroid medication before the pregnancy. The study analysis was conducted by both including and excluding these mothers.

The trimester-specific reference intervals for TSH and fT4 were 0.07–3.1 mU/L and 11.4–22.4 pmol/L in the first trimester and 0.10–3.5 mU/L and 11.09–18.9 pmol/L in the second trimester (34). Mothers with a serum TPO-Ab concentration over the 95th percentile (>167.7 IU/mL) were categorized as TPO-Ab-positive (5.1% of mothers) (34).

Mothers with parental questionnaire report data were divided into four groups according to their serum fT4 and TSH concentrations:

(1) Euthyroidism: maternal TSH and fT4 within the reference intervals, *n* = 4,831.
(2) Hypothyroidism: TSH above the upper reference interval with low or normal fT4 concentrations, *n* = 365.
(3) Hypothyroxinemia: TSH within the reference intervals with low fT4 concentrations, *n* = 71.
(4) Hyperthyroidism: TSH below the lower reference interval with high or normal fT4 concentrations, *n* = 124.

The number of mothers with thyroid dysfunction varied in different analyses due to missing questionnaires and/or clinical examination data.

### Final Study Population

We excluded mothers with multiple pregnancies (*n* = 232), mothers who refused use of data (*n* = 251), mothers with insufficient or missing samples for thyroid function analyses (*n* = 3,018), and mothers with a gestational age at sampling >20 weeks (*n* = 187). We also excluded children with an intelligence quotient (IQ) ≤85 (*n* = 147) because they were previously reported to have congenital syndromes and disorders that affect overall development such as Down syndrome which lead to 5,644 mother–child pairs. After excluding those with missing questionnaire data the final study population in sensory and linguistic development analyses was 5,391 mother–child pairs. The percentage of questionnaire completion in the final sample varied (56.3–100%) depending on the questions.

### Statistical Analysis

Maternal and family characteristics of mothers in thyroid dysfunction groups were compared with euthyroid mothers *via t*-tests for continuous variables with normal distribution. Mann–Whitney *U* tests were used for those with non-Gaussian distributions. Categorical variables were compared *via* χ^2^ tests.

Study outcomes were dichotomized. We studied the composite outcomes of any vision defects, abnormal speech development, and hearing defects as well as the prevalence of a specific defect. To assess if median maternal TSH, fT4, and TPO-Ab concentrations differed among mothers with and without questionnaire data, the Mann–Whitney *U* test was used. The prevalence of abnormal questionnaire results from mothers with and without the thyroid dysfunction and with or without positive TPO-antibody concentrations was evaluated with a χ^2^ test. Odds ratios (ORs) with 95% CIs for children with any visual impairment, abnormal faculty of speech, or hearing defect were assessed with logistic regression. No further adjustments were made, because all raw results were statistically non-significant.

All data were analyzed by including and excluding TPO-Ab-positive mothers and mothers with diagnosed and treated thyroid disease during or prior to the index pregnancy. Since the results did not differ, all of these mothers were included in the final analysis and presented results. The data were stratified to term and preterm children to see if preterm birth modified the association.

All statistical analyses were performed using SPSS version 20.0 software (IBM Statistics).

## Results

Table 1 presents the demographic characteristics of the NFBC 1986 mothers during their index pregnancies. Hypothyroid mothers had a higher pre-pregnancy body mass index and smoked less often than euthyroid mothers. Hyperthyroid mothers were older than euthyroid mothers. Hypothyroxinemic mothers had higher pre-pregnancy body mass index and were older than euthyroid mothers (Table 1). TPO-Ab-positivity was the most prevalent in the group of hypothyroid mothers (113/365, 31%) (Table 1). The median maternal TSH, fT4, and TPO-Ab concentrations did not differ between mothers with and without sensory development data on the child (Table 2). The prevalence of child impairments did not significantly differ between mothers with and without laboratory data. Table 3 presents the respondent percentages in different question categories.

**Table 1:** Maternal and family characteristics grouped by maternal thyroid function.

Characteristics	Euthyroid (*n* = 4,831/160)	Hypothyroid (*n* = 365/113)	Hyperthyroid (*n* = 124/3)	Hypothyroxinemic (*n* = 71/5)
Median (IQR) maternal TSH concentration, mU/L	1.2 (0.7–1.7)	4.1 (3.6–5.4)*	0.03 (0.02–0.04)*	1.3 (0.9–2.0)
Median (IQR) maternal free T4, pmol/L	15.1 (13.8–16.5)	13.9 (12.5–15.2)*	20.5 (18.0–22.9)*	11.0 (10.7–11.2)*
Median (IQR) maternal TPO-Ab concentration, IU/mL	4.2 (3.0–6.2)	27.7 (5.0–321.6)*	4.3 (3.2–6.1)	3.5 (2.3–5.6)*
Mean (SD) maternal age at birth, years	28.1 (5.3)	28.4 (5.4)	30.0 (5.5)*	30.2 (6.2)*
Mean (SD) BMI, kg/m^2^	22.0 (3.3)	22.6 (3.6)*	22.4 (3.4)	23.9 (5.0)*
Overweight/obese (BMI > 25 kg/m^2^)	739 (15.9)	71 (20.1)	22 (18.6)	16 (26.1)*
**Maternal education**				
≥11 years, *n* (%)	2,604 (61.5)	187 (58.8)	67 (59.3)	34 (54.0)
<11 years, *n* (%)	1,633 (38.5)	131 (41.2)	46 (40.7)	29 (46.0)
Smoking during pregnancy, *n* (%)	1,030 (21.4)	54 (14.9)*	19 (15.4)	17 (24.3)
Socioeconomic status of the family				
Professional, *n* (%)	2,817 (79.1)	199 (78.7)	76 (80.9)	42 (85.7)
Skilled, *n* (%)	581 (16.3)	44 (17.4)	12 (12.8)	4 (8.2)
Unskilled, *n* (%)	26 (0.7)	4 (1.6)	0	1 (2.0)
Farmers, *n* (%)	137 (3.8)	6 (2.4)	6 (6.4)	2 (4.1)
Mean (SD) gestational age at maternal serum sampling, weeks	10.7 (2.8)	10.7 (2.7)	10.9 (2.5)	10.7 (2.8)
Preterm births (<37 weeks), *n* (%)	207 (4.3)	17 (4.7)	5 (4.0)	6 (8.5%)
Male children, *n* (%)	2,454 (50.8)	194 (53.2)	44 (35.5)*	42 (59.2)

*The groups include mothers with child sensory development data available and child intelligence quotient >85*.

*n = number of all mothers in the thyroid function group/number of TPO-Ab-positive mothers*.

*Euthyroidism: maternal TSH and fT4 both within the reference intervals. Hypothyroidism: TSH above its upper limit with low or normal fT4 concentrations. Hyperthyroidism: TSH below its lower reference limit with high or normal fT4 concentrations. Hypothyroxinemia: TSH within the reference intervals with low fT4 concentrations*.

*p < 0.05, when the maternal thyroid dysfunction group was compared with euthyroid mothers: *t*-test or Mann–Whitney *U* test (continuous variables), χ^2^ test (categorical variables).

*TSH, thyrotropin; fT4, free thyroxine; TPO-Ab, thyroid peroxidase antibodies; BMI, body mass index; IQR, interquartile range*.

**Table 2:** Maternal thyroid function values, grouped by children with and without parent-reported questionnaire data.

Maternal thyroid function parameter	Sensory function data available (*n* = 5,391/281*)	Sensory function data not available (*n* = 414/9**)
TSH (mU/L)	1.20 (0.20–3.60)	1.20 (0.18–3.54)
fT4 (pmol/L)	15.04 (11.9–20.55)	15.13 (12.0–20.52)
TPO-Ab (IU/mL)	4.18 (2.07–162.06)	4.28 (2.02–175.43)

n = number of all mothers/TPO-Ab-positive mothers. TPO-Ab-analysis missing from *1 and **37% of the mothers. Data expressed as median (5–95th percentile). Children with an intelligence quotient ≤85 excluded from analysis.

**Table 3:** Parental questionnaire response percentage.

Question category	*n* of answered questions/*n* of parental questionnaires (%)
Vision	3,985/5,391 (74%)
Speech	4,795–5,054/5,391 (89.0–93.7%)
Hearing	3,035–5,391/5,391 (56.3–100%)

*Children with maternal thyroid function data available and intelligence quotient >85 included in the analysis, total n = 5,391*.

Table 4 presents the parental reports of abnormal results for sensory development, grouped by maternal thyroid function status. The prevalence of adverse outcomes in children of mothers with thyroid dysfunction did not statistically differ from that of euthyroid mothers. Children of hypothyroid and hypothyroxinemic mothers had a higher prevalence of any vision deficit (10.8 and 11.7%, respectively) than those of euthyroid mothers (6.5%). However, this difference was not statistically significant. The respective ORs (95% CIs) were 1.39 (0.90–2.14) for the children of hypothyroid mothers and 1.89 (0.85–4.20) for hypothyroxinemic mothers. Children of TPO-Ab-positive mothers did not have statistically significantly more often problems in any outcome categories.

**Table 4:** Child’s parent-reported sensory development, grouped by maternal thyroid function during early pregnancy.

	Maternal thyroid function group				
Child’s sensory outcome	Euthyroid (*n* = 4,831)	Hypothyroid (*n* = 365)	Hypothyroxinemic (*n* = 71)	Hyperthyroid (*n* = 124)	Total (*n* = 5,391)
**Vision**					
Squint	152/3,564 (4.3%)	16/282 (5.7%)	4/60 (6.7%)	2/79 (2.5%)	174/3,985 (4.3%)
Refractive error	135/3,564 (3.8%)	14/282 (5.0%)	4/60 (6.7%)	3/79 (3.8%)	156/3,985 (3.9%)
Other	20/3,564 (0.6%)	4/282 (1.4%)	1/60 (1.7%)	1/79 (1.3%)	26/3,985 (0.7%)
Any vision defect	233/3,564 (6.5%)	25/282 (10.8%)	7/60 (11.7%)	4/79 (5.1%)	269/3,985 (6.7%)

**Speech**					
Is your child’s way of speech normal	385/4,529 (8.5%)	24/345 (7.0%)	8/64 (12.5%)	10/116 (8.6%)	427/5,054 (8.4%)
Delayed development of speech or dysphasia	102/4,317 (2.4%)	7/331 (2.1%)	1/59 (1.7%)	1/113 (0.9%)	111/4,820 (2.3%)
Other	212/4,311 (4.9%)	10/330 (3.0%)	4/59 (6.8%)	7/113 (6.2%)	233/4,813 (4.8%)
Needed speech therapy, under 4 years old	110/4,294 (2.6%)	6/329 (1.8%)	1/59 (1.7%)	0/113	117/4,795 (2.4%)
Any speech deficit	477/4,538 (10.5%)	30/345 (8.7%)	9/64 (14.1%)	10/116 (8.6%)	526/5,063 (10.3%)

**Hearing**					
Lowered hearing ability	87/4,527 (1.9%)	4/345 (1.2%)	1/64 (1.6%)	2/116 (1.7%)	94/5,052 (1.8%)
Examined for abnormal hearing	92/2,698 (3.4%)	12/217 (5.5%)	0/64	3/56 (5.4%)	107/3,035 (3.5%)
Abnormal hearing in either ear	120/4,831 (2.5%)	10/365 (2.7%)	1/71 (1.4%)	2/124 (1.6%)	133/5,391 (2.5%)
Any hearing deficit	252/4,831 (5.2%)	22/365 (6.0%)	2/71 (2.8%)	4/124 (3.2%)	280/5,391 (5.2%)

*Children with maternal thyroid function data available and intelligence quotient >85 included in the analysis, total *n* = 5,391. Prevalence of abnormal questionnaire results in children of mothers with and without thyroid dysfunction (euthyroid group as reference group) was evaluated with a χ^2^ test. No statistically significant results were found*.

*Euthyroidism: maternal TSH and fT4 both within the reference intervals. Hypothyroidism: TSH above its upper limit with low or normal fT4 concentrations. Hyperthyroidism: TSH below its lower reference limit with high or normal fT4 concentrations. Hypothyroxinemia: TSH within the reference intervals with low fT4 concentrations*.

All results remained similar after excluding mothers with positive TPO-Ab concentrations and prematurely born children.

## Discussion

In this study, no statistically significant association between maternal thyroid dysfunction during early pregnancy and childhood vision, hearing, or speech development were found. We did not find previous large-scale studies examining maternal thyroid dysfunction during pregnancy and subsequent overall childhood sensory development.

Mild visual impairment is a relatively common childhood problem, but severe visual impairments or blindness are rare and often accompanied by varying comorbidities (35). Children at the highest risk of visual impairment are those born preterm, or with cerebral palsy, brain injury, learning difficulty, Down syndrome, or hearing impairment (35). We excluded children with and IQ ≤85 to attenuate confounding by these factors. We found no significant association between maternal hypothyroidism or hypothyroxinemia during pregnancy and parent-reported vision impairment in children. A slight tendency toward increased visual impairment was seen among children of hypothyroxinemic mothers although this did not reach statistical significance. One previous study found that untreated overt maternal hypothyroidism associated with reduced contrast sensitivity in children (18). It is plausible that severe maternal thyroid dysfunction associates with visual impairment as vision development is sensitive to disturbances (36) and rodent studies have shown that THs and normal TH metabolism during fetal period are needed for normal vision (15–17, 37). This is also in line with the previous knowledge of adverse child outcomes associated with overt maternal hypothyroidism (2). The effects of milder forms of maternal thyroid dysfunction during pregnancy on vision development remain unclear.

Thyroid hormones are also important for audiological development as type 3 deiodinase deficient mice suffer deafness and premature cochlear differentiation (38). In this study, we found no connection between maternal thyroid dysfunction and hearing problems in children. In one previous study, maternal third trimester TPO-Ab-positivity was associated with an increased risk of sensorineural hearing loss in children at 8 years (26). However, maternal TSH and fT4 concentrations were not evaluated in that study. Accounting for maternal TPO-Ab positivity did not change our findings, suggesting little effect of TPO-Ab positivity on hearing problems in children in our cohort. Childhood hearing impairment has a prevalence of 2.3/1,000 in Northern Finland and the etiology is 47% genetic, 16% acquired, and 36% unknown (39). We accounted for these factors by excluding children with an IQ ≤85, because the most common etiology behind intellectual impairment in the NFBC 1986 was a disease or syndrome (40). We also excluded children born preterm, since asphyxia and prematurity are currently most common etiologies for hearing impairment (41), but this had little effect on our results.

Speech disorders and language delays are common multifactorial childhood problems, with an estimated prevalence of up to 12% (42). Speech disorders have been previously studied in the NFBC 1986 in children with low-birth weight (<2,500 g), who had an increased risk of poorer linguistic skills compared with children with normal birth weight (43). In the present study, maternal thyroid dysfunction was not associated with childhood speech abnormalities. Our findings contradict those of Henrichs et al. who associated maternal hypothyroxinemia with an increased risk of expressive language delay in children at 18 and 30 months (25). Since our measurement of speech impairment was geared toward capturing conditions that require treatment such as speech therapy, we may have missed more subtle differences in language development such as those reported by Henrichs et al.

One strength of the present study is the population-based cohort setting, covering almost the entire Northern Finland. Another strength is the communal child welfare clinic system, which assures that all cohort children were examined identically during follow-up. The maternal serum samples were mainly drawn in the first trimester and studied in a single laboratory. The comprehensive cohort data collection from pregnancy onward permitted us to categorize children into term and preterm births and exclude those with an IQ ≤85. A recent publication showed that pregnant women are particularly at risk of iodine deficiency (44). Iodine insufficiency during pregnancy should not confound our results because iodine supplementation has been in use in Finland since the 1940s (45, 46). In 1986, Finland had the highest iodine intake of all European countries, approximately 300 μg/day (47). At that time, Finland was considered to be iodine sufficient.

As limitations, we lacked some data on maternal thyroid function assessments. However, we previously confirmed that mothers with and without laboratory data do not significantly differ (33). We also lacked some parental evaluations of their children’s sensory development, though the overall parental questionnaire response rate was excellent (90%). Questions on the child’s vision were answered less often. Nevertheless, due to the comprehensive screening system, parental evaluations most likely relied on welfare clinic examinations. Presumably, parents would remember the most important findings from the screening visits. However, the clinical examinations in the screening program together with the parental questionnaire were not the most sensitive method to distinguish the subtlest changes in vision and visual processing development between children of mothers in different thyroid function groups. That would require a different study setting. Although our cohort data have been collected almost a generation ago, the screening system has remained the same in Finland. Our material was large, but certain rare outcomes were difficult to reliably study in small maternal thyroid dysfunction groups.

In conclusion, maternal thyroid dysfunction during pregnancy did not affect childhood sensory and linguistic development in our cohort study.

## Ethics Statement

This study was carried out in accordance with the recommendations of the Ethics Committees of the Northern Ostrobothnia Hospital District, Finland, and the National Institute for Health and Welfare, Finland. All subjects gave written informed consent in accordance with the Declaration of Helsinki. The protocol was approved by the Ethics Committees of the Northern Ostrobothnia Hospital District, Finland.

## Author Contributions

FP was the first author responsible for data analysis of and manuscript writing. TM was the main instructor concerning data analysis and writing. ES took part especially in study design planning, research plan writing and manuscript introduction, and discussion writing. A-LH took part in the whole cohort study planning and executing, data collection and storing, and also helped with planning and finalizing this current manuscript.

## Conflict of Interest Statement

The authors declare that the research was conducted in the absence of any commercial or financial relationships that could be construed as a potential conflict of interest.
